# Supplementary material for: Protective Effects of Early Caffeine Administration in Hyperoxia-Induced Neurotoxicity in the Juvenile Rat
Source: Antioxidants (Basel). 2023 Jan 28;12(2):295. doi: 10.3390/antiox12020295 (PMC9952771; doi:10.3390/antiox12020295)
Supplement: Supplementary file 1 [file antioxidants-12-00295-s001.zip › Fig S-1 Legend to Experimental design.pdf]

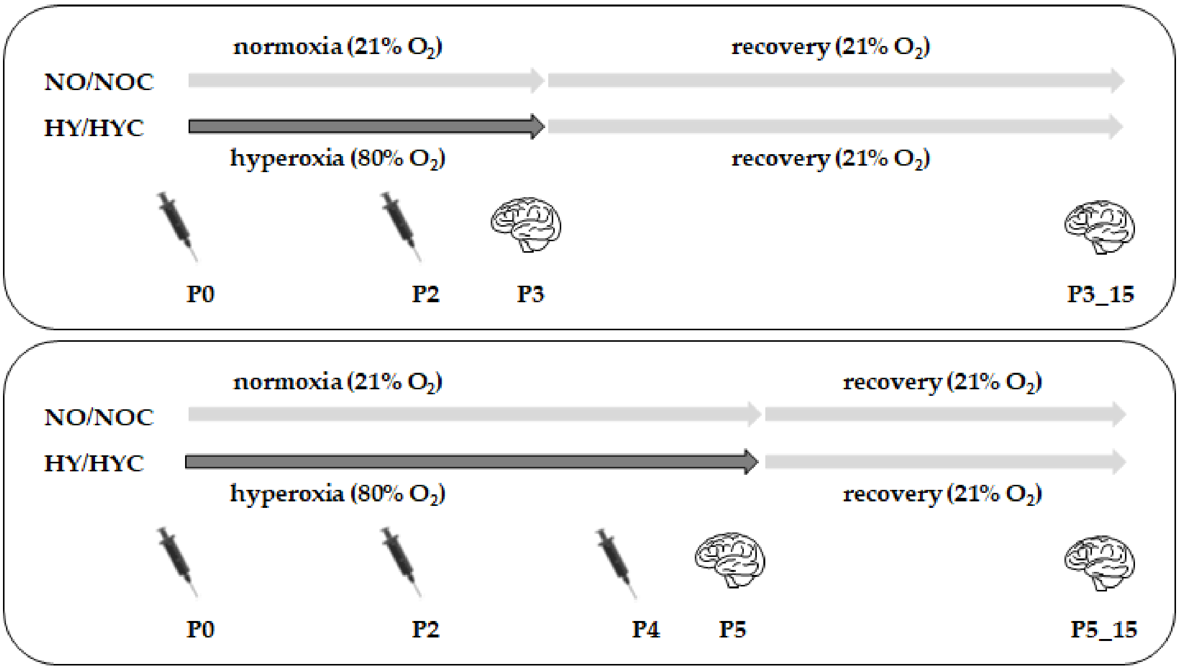

Figure S1. Experimental design. Oxygen exposure was performed in parallel with 21% or 80% oxygen from postnatal day (P)0 to P3 (n=6−8) or P0 to P5 (*n*=6−8). Keeping the same exposure times, rats were further divided into four groups: i) normoxic (NO, control group): 21% oxygen with vehicle (phosphate−buffered saline, PBS), ii) normoxia with caffeine (NOC): 21% oxygen with caffeine (10 mg/kg), iii) hyperoxia (HY): 80% oxygen with vehicle (PBS), and iv) hyperoxia with caffeine (HYC): 80% oxygen with caffeine (10 mg/kg). Applications were intraperitoneally (i.p.) injected as a fixed fraction of their body weight (100 µl/10 g) every 48 hours starting from day of birth (P0). The administration of caffeine or vehicle took place for the pups with a total of three postnatal days of oxygen exposure (P0−P3) on the day of birth (P0) and on P2; for the rat pups with a total of five days of postnatal oxygen exposure (P0−P5) on the day of birth (P0) and on P2 and P4. Rat pups were examined after exposure to oxygen (P3, P5) directly or after recovery in room air at P15 (P3_15, P5_15).
