# Supplementary material for: Protective Effects of Early Caffeine Administration in Hyperoxia-Induced Neurotoxicity in the Juvenile Rat
Source: Antioxidants (Basel). 2023 Jan 28;12(2):295. doi: 10.3390/antiox12020295 (PMC9952771; doi:10.3390/antiox12020295)
Supplement: Supplementary file 1 [file antioxidants-12-00295-s001.zip › Fig S-2 Legend to Quantification of DAPI counts.pdf]

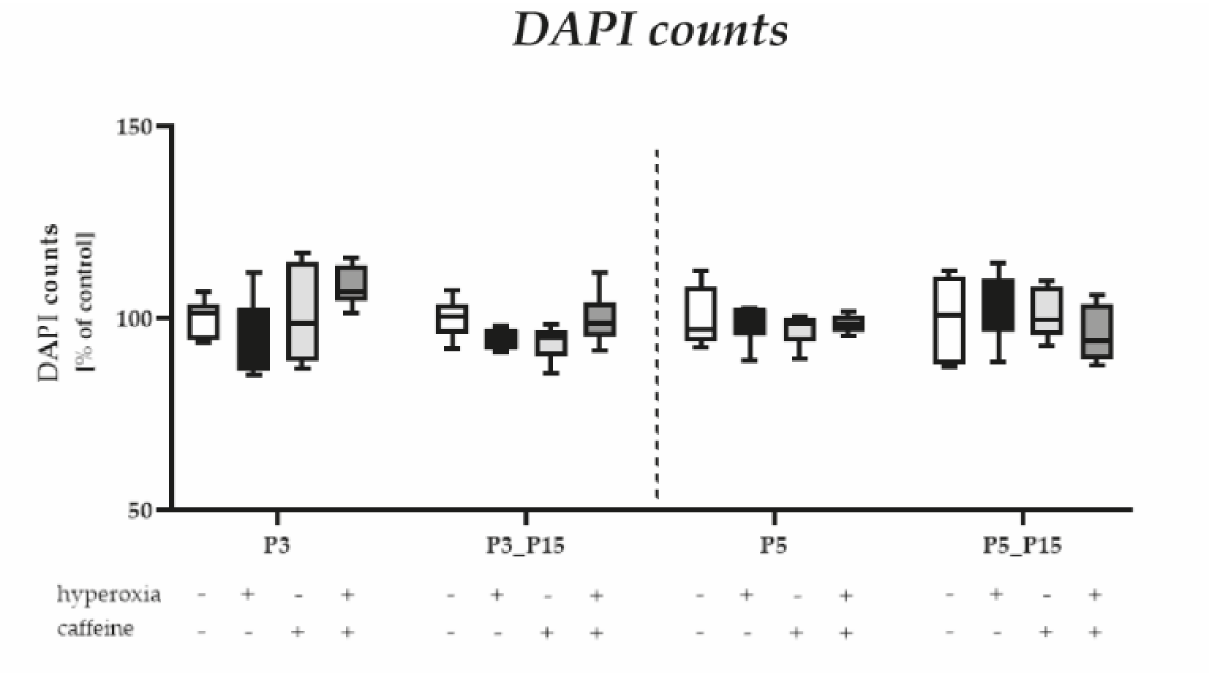

Figure S2. Quantification of counts of labeled DAPI positive hippocampal cells are presented as hyperoxia exposure (black bars), hyperoxia in combination with caffeine administration (deep gray bars), and caffeine with normoxia (light gray bars) in comparison to normoxia vehicle−treated control animals (white bars). Three (P3) and five days (P5) lasting hyperoxia as well as after recovery until P15 (P3_15, P5_15) doesn´t affect the proportion of DAPI positive cells. Data are normalized to the level of rat pups exposed to normoxia at each time point (control 100 %, white bars) and the 100 % values are 979.7 (P3), 1090.7 (P3_15), 1050.1 (P5), and 1070.7 (P5_15) for DAPI positive cells. n = 5.6/ group (ANOVA, Bonferroni's post hoc test; Kruskal−Wallis, Dunn´s post hoc test; Brown−Forsythe, Dunnett´s post hoc test).
