# Supplementary material for: Protective Effects of Early Caffeine Administration in Hyperoxia-Induced Neurotoxicity in the Juvenile Rat
Source: Antioxidants (Basel). 2023 Jan 28;12(2):295. doi: 10.3390/antiox12020295 (PMC9952771; doi:10.3390/antiox12020295)
Supplement: Supplementary file 1 [file antioxidants-12-00295-s001.zip › Tab S-1 Quantitation of NeuroD1 and PCNA Counts.pdf]

Table S1. Quantitation of NeuroD1 and PCNA positive cells after oxygen−induced cerebral neurotoxicity with/without caffeine

**hyperoxia**	−	+	−	+	**hyperoxia**	−	+	−	+
**caffeine**	−	−	+	+	**caffeine**	−	−	+	+
		**P3**					**P3_15**		
**NeuroD1/PCNA**	100±4.7	69±3.6	100±4.1	107±7.4	**NeuroD1/PCNA**	100±12.2	62±3.7	54±5.3	82±2.7
**PCNA**	100±2.3	71±7.9	85±9.4	108±5.2	**PCNA**	100±8.9	59±4,8	50±6,9	81±7.8
		**P5**					**P5_15**		
**NeuroD1/PCNA**	100±12.4	26±4.9	95±5.4	155±8.8	**NeuroD1/PCNA**	100±13.1	64±9.3	101±8.2	145±6.8
**PCNA**	100±8.9	34±3.2	77±8.1	122±7.9	**PCNA**	100±6.9	33±2.7	41±4.2	65±5.9

Data are normalized to the level of rat pups exposed to normoxia at each time point (control 100 %,) and the 100 % values are 71.8 (P3), 84.1 (P3_15), 85.6 (P5), and 52.9 (P5_15) for NeuroD1/PCNA positive cells, or 157.9 (P3), 407.1 (P3_15), 391.9 (P5), and 249.3 (P5_15) for PCNA positive cells, respectively. Data expressed as % of control as mean ± SEM with n = 5−6/ group. The significant values can be obtained from the diagrams in Fig. 2.
