# Supplementary material for: Protective Effects of Early Caffeine Administration in Hyperoxia-Induced Neurotoxicity in the Juvenile Rat
Source: Antioxidants (Basel). 2023 Jan 28;12(2):295. doi: 10.3390/antiox12020295 (PMC9952771; doi:10.3390/antiox12020295)
Supplement: Supplementary file 1 [file antioxidants-12-00295-s001.zip › Tab S-2 Quantitation of NeuN Counts.pdf]

Table S2. Quantitation of NeuN positive cells after oxygen−induced cerebral neurotoxicity with/without caffeine

**hyperoxia**	−	+	−	+	**hyperoxia**	−	+	−	+
**caffeine**	−	−	+	+	**caffeine**	−	−	+	+
		**P3**					**P3_15**		
**NeuN**	100±5.2	77±4.9	95±10.3	109±4.2	**NeuN**	100±5.2	77±4.9	95±10.3	109±4.2
**DAPI**	100±2.0	95±3.7	101±5.4	108±2.2	**DAPI**	100±2.1	95±1.2	94±1.9	100±2.8
		**P5**					**P5_15**		
**NeuN**	100±5.2	77±4.9	95±10.3	109±4.2	**NeuN**	100±5.2	77±4.9	95±10.3	109±4.2
**DAPI**	100±3.3	99±2.2	97±1.7	98±1.0	**DAPI**	100±4.6	103±3.7	101±2.7	96±3.4

Data are normalized to the level of rat pups exposed to normoxia at each time point (control 100 %) and the 100 % values are 511.1 (P3), 480.7 (P3_P15), 590.1 (P5), and 461.4 (P5_P15) for NeuN positive cells, or 979.7 (P3), 1090.7 (P3_15), 1050.1 (P5), and 1070.7 (P5_15) for DAPI positive cells, respectively. Data expressed as % of control as mean ± SEM with n = 5−6/ group. The significant values can be obtained from the diagrams in Fig. 3 (NeuN) and Fig. S−2 (DAPI).
