# Supplementary material for: Protective Effects of Early Caffeine Administration in Hyperoxia-Induced Neurotoxicity in the Juvenile Rat
Source: Antioxidants (Basel). 2023 Jan 28;12(2):295. doi: 10.3390/antiox12020295 (PMC9952771; doi:10.3390/antiox12020295)
Supplement: Supplementary file 1 [file antioxidants-12-00295-s001.zip › Tab S-3 Quantitation of Neural Progenitor-associated mRNA Expression.pdf]

Table S3. Quantitation of neural progenitor−associated mRNA expression after oxygen−induced cerebral neurotoxicity with/without caffeine

**hyperoxia**	**−**	**+**	**−**	**+**	**hyperoxia**	**−**	**+**	**−**	**+**
**caffeine**	**−**	**−**	**+**	**+**	**caffeine**	**−**	**−**	**+**	**+**
**P3**					**P3_15**				
Scl1a3	100±6.7	103±5.7	90±7.6	113±6.0	*Scl1a3*	100±3.7	141±7.6	102±3.7	101±6.3
GFAP	100±5.6	111±5.7	96±8.9	88±8.1	*GFAP*	100±3.7	100±4.8	94±4.9	95±2.5
Hes5	100±6.2	105±8.2	85±4.1	86±5.5	*Hes5*	100±6.9	133±5.6	92±5.9	80±4.9
Pax6	100±6.5	68±4.8	60±4.2	53±3.1	*Pax6*	100±8.2	132±8.8	99±5.0	96±6.6
Sox2	100±7.0	96±9.3	50±6.6	22±2.8	*Sox2*	100±4.3	120±7.5	86±4.9	30±3.9
**P5**					**P5_15**				
Scl1a3	100±7.3	59±5.4	80±6.9	140±7.6	*Scl1a3*	100±4.2	77±3.2	82±6.3	74±7.0
GFAP	100±5.8	95±2.3	49±4.5	111±12.9	*GFAP*	100±7.5	103±2.2	91±7.3	96±7.1
Hes5	100±3.2	68±2.5	85±5.3	140±13.1	*Hes5*	100±5.1	58±2.9	95±5.3	94±8.9
Pax6	100±5.9	79±6.7	160±10.3	158±7.0	*Pax6*	100±3.6	103±2.8	107±6.4	159±11.1
Sox2	100±8.1	60±5.7	67±7.5	105±4.4	*Sox2*	100±3.7	93±3.2	94±8.2	104±8.5

Data are normalized to the level of rat pups exposed to normoxia at each time point (control 100 %). Data expressed as % of control as mean ± SEM with n = 7–8/ group. The significant values can be obtained from the diagrams in Fig. 4.
