# Supplementary material for: Protective Effects of Early Caffeine Administration in Hyperoxia-Induced Neurotoxicity in the Juvenile Rat
Source: Antioxidants (Basel). 2023 Jan 28;12(2):295. doi: 10.3390/antiox12020295 (PMC9952771; doi:10.3390/antiox12020295)
Supplement: Supplementary file 1 [file antioxidants-12-00295-s001.zip › Tab S-4 Quantitation of Intermediate Neuronal Progenitor-Cell-associated mRNA Expression.pdf]

Table S4. Quantitation of intermediate neuronal progenitor−cell−associated mRNA expression after oxygen−induced cerebral neurotoxicity with/without caffeine

**hyperoxia**	−	+	−	+	**hyperoxia**	−	+	−	+
**caffeine**	−	−	+	+	**caffeine**	−	−	+	+
		**P3**					**P3_15**		
Ascl1	100±5.4	70±3.0	45±4.7	66±4.4	*Ascl1*	100±7.5	145±7.6	60±2.6	69±4.2
Ngn2	100±5.6	58±4.9	61±5.7	81±6.3	*Ngn2*	100±5.8	69±4.9	53±4.2	92±6.3
Tbr2	100±8.9	72±7.4	53±6.0	44±7.8	*Tbr2*	100±8.8	55±3.5	96±12.7	87±10.3
NeuroD1	100±6.6	62±6.1	103±12.9	120±11.4	*NeuroD1*	100±5.9	133±8.7	[b]55±2.6	57±1.9
CycD2	100±7.4	66±5.1	95±6.7	125±7.9	*CycD2*	100±5.2	99±5.1	101±8.1	134±5.1
		**P5**					**P5_15**		
Ascl1	100±3.9	69±6.5	76±7.6	105±9.6	*Ascl1*	100±6.9	92±5.2	65±4.2	80±7.2
Ngn2	100±8.7	120±19.0	95±11.8	93±11.2	*Ngn2*	100±8.9	156±14.9	59±8.6	67±12.5
Tbr2	100±4.9	67±7.9	74±8.1	152±14.8	*Tbr2*	100±9.5	73±6.2	63±8.1	95±10.2
NeuroD1	100±7.9	71±11.2	59±6.5	125±16.1	*NeuroD1*	100±4.8	131±3.9	93±6.5	77±4.8
CycD2	100±3.7	65±1.9	90±13.0	119±8.6	*CycD2*	100±5.8	70±2.7	69±6.4	92±6.2

Data are normalized to the level of rat pups exposed to normoxia at each time point (control 100 %). Data expressed as % of control as mean ± SEM with n = 7–8/ group. The significant values can be obtained from the diagrams in Fig. 5.
