# Supplementary material for: Protective Effects of Early Caffeine Administration in Hyperoxia-Induced Neurotoxicity in the Juvenile Rat
Source: Antioxidants (Basel). 2023 Jan 28;12(2):295. doi: 10.3390/antiox12020295 (PMC9952771; doi:10.3390/antiox12020295)
Supplement: Supplementary file 1 [file antioxidants-12-00295-s001.zip › Tab S-5 Quantitation of Mature Granular Neuron-associated mRNA Expression .pdf]

Table S5. Quantitation of mature granular neuron−associated mRNA expression after oxygen−induced cerebral neurotoxicity with/without caffeine

**hyperoxia**	−	+	−	+	**hyperoxia**	−	+	−	+
**caffeine**	−	−	+	+	**caffeine**	−	−	+	+
		**P3**					**P3_15**		
NeuroD2	100±5.9	52±2.1	62±7.2	72±4.7	*NeuroD2*	100±6.2	70±2.7	62±2.9	79±6.9
Prox1	100±4.5	69±5.6	70±7.3	145±14.8	*Prox1*	100±6.0	106±8.2	51±4.9	61±5.9
Tbr1	100±6.8	71±1.8	79±1.5	99±7.1	*Tbr1*	100±5.6	125±4.8	82±7.3	94±4.5
		**P5**					**P5_15**		
NeuroD2	100±7.3	60±8.2	72±9.1	130±10.3	*NeuroD2*	100±6.8	98±6.5	69±7.2	71±7.0
Prox1	100±5.4	59±4.9	98±8.3	171±17.9	*Prox1*	100±5.4	144±5.7	75±8.3	68±6.2
Tbr1	100±9.7	55±8.4	61±9.0	103±9.3	*Tbr1*	100±5.8	71±3.6	79±4.7	114±3.9

Data are normalized to the level of rat pups exposed to normoxia at each time point (control 100 %). Data expressed as % of control as mean ± SEM with n = 7−8/ group. The significant values can be obtained from the diagrams in Fig. 6.
