# Supplementary material for: Protective Effects of Early Caffeine Administration in Hyperoxia-Induced Neurotoxicity in the Juvenile Rat
Source: Antioxidants (Basel). 2023 Jan 28;12(2):295. doi: 10.3390/antiox12020295 (PMC9952771; doi:10.3390/antiox12020295)
Supplement: Supplementary file 1 [file antioxidants-12-00295-s001.zip › Tab S-6 Quantitation of Neurotrophin-associated mRNA Expression.pdf]

Table S6. Quantitation of neurotrophin−associated mRNA expression after oxygen−induced cerebral neurotoxicity with/without caffeine

**hyperoxia**	−	+	−	+	**hyperoxia**	−	+	−	+
**caffeine**	−	−	+	+	**caffeine**	−	−	+	+
		**P3**					**P3_15**		
BDNF	100±6.3	60±1.7	97±10.8	146±12.9	*BDNF*	100±4.0	131±6.9	82±3.8	69±1.9
NGF	100±4.7	72±3.5	107±6.9	104±5.1	*NGF*	100±5.6	139±5.9	84±4.9	81±3.6
NT3	100±6.7	58±5.4	93±8.2	188±23.7	*NT3*	100±6.8	162±8.0	71±3.2	72±4.9
		**P5**					**P5_15**		
BDNF	100±7.7	53±4.5	106±6.3	144±13.6	*BDNF*	100±4.9	128±3.4	117±9.9	83±9.8
NGF	100±6.2	98±6.3	94±5.0	159±8.1	*NGF*	100±8.9	103±7.8	66±6.4	108±6.5
NT3	100±6.9	66±6.4	59±3.0	130±15.2	*NT3*	100±7.4	163±6.9	61±6.2	79±7.4

Data are normalized to the level of rat pups exposed to normoxia at each time point (control 100 %). Data expressed as % of control as mean ± SEM with n = 7−8/ group. The significant values can be obtained from the diagrams in Fig. 7.
